# The proton pump inhibitor, lansoprazole, prevents the development of non-traumatic osteonecrosis of the femoral head: an experimental and prospective clinical trial

**DOI:** 10.1007/s00590-020-02622-5

**Published:** 2020-01-14

**Authors:** Ima Kosukegawa, Shunichiro Okazaki, Motohisa Yamamoto, Satoshi Nagoya, Chisako Suzuki, Junya Shimizu, Hiroki Takahashi, Toshihiko Yamashita

**Affiliations:** 1 Department of Orthopedic Surgery, Sapporo Medical University School of Medicine, S1 W16, Chuo-ku, Sapporo, 060-8543 Japan; 2 Department of Orthopedic Surgery, Hokkaido Ohno Memorial Hospital, Sapporo, Japan; 3 Department of Rheumatology and Allergy, The Institute of Medical Science, The University of Tokyo, Tokyo, Japan; 4 Department of Musculoskeletal Biomechanics and Surgical Development, Sapporo Medical University School of Medicine, Sapporo, Japan; 5 Department of Rheumatology and Clinical Immunology, Sapporo Medical University School of Medicine, Sapporo, Japan

**Keywords:** Osteonecrosis of the femoral head, Prevention, Lansoprazole, Clinical trial, Experimental animal model

## Abstract

**Background:**

An effective prevention strategy for osteonecrosis of the femoral head (ONFH) has yet to be established. We previously reported that the innate immune system via the toll-like receptor (TLR) response induced by corticosteroids leads to the development of ONFH and that repression of IRF7 activity by an inhibitor could interfere with the development of ONFH while maintaining the therapeutic effect of the corticosteroids.

**Objective:**

In the present study, we hypothesize that lansoprazole has the potential to suppress IRF7 activity and prevent corticosteroid-induced ONFH in rats. Furthermore, we conducted a preliminary clinical trial to prevent corticosteroid-induced ONFH in autoimmune disease patients.

**Methods:**

Male Wistar rats were randomly divided into four groups. On Day 1, each rat was injected with TLR4 ligand (LPS) or TLR7 ligand (imiquimod), followed by methylprednisolone with or without lansoprazole on Day 2. They were killed at 1 or 14 days after the last injection.We prospectively recruited 30 patients requiring primary high-dose corticosteroid treatment for immune diseases. All patients were administered lansoprazole, starting the night before corticosteroid treatment began. MRI was performed before corticosteroid treatment, and at 4, 12 and 24 weeks afterward.

**Results:**

In rats, co-treatment of lansoprazole with corticosteroids significantly repressed both IRF7 activity and the development of ONFH. Moreover, in the human patients, the incidence of ONFH was significantly decreased from 53.4 to 13.3%.

**Conclusions:**

Although the present study is preliminary, the results show that co-treatment of lansoprazole with corticosteroids prevents ONFH development. Lansoprazole may be both safe and effective in preventing osteonecrosis of the femoral head in patients needing corticosteroid treatment.

## Introduction

High-dose corticosteroid therapy for inflammatory diseases and alcohol-abuse was reported to be a risk factor for non-traumatic osteonecrosis of the femoral head (ONFH) [1–3]. The pathogenesis of ONFH in patients with inflammatory diseases treated with corticosteroids remains unclear, although there has reported as factors caused ischemia due to fat emboli in peripheral blood vessels, reduction in arterial flow through the recruitment of increased adipose tissue and/or an increase in intramedullary pressure [4, 5]. On the other hand, ONFH frequently results in osteoarthritis in young patients [6, 7], and no effective prevention strategy or effective nonsurgical treatment for ONFH has been established to date.

We previously reported corticosteroid-induced ONFH rat models treated with a toll-like receptor (TLR) ligand and corticosteroid and that TLR signaling pathways contribute to the pathogenesis of corticosteroid-induced ONFH in rats [8, 9]. We also reported an alcohol-induced ONFH rat model and that the TLR4 signaling pathway contributes to the pathogenesis of alcohol-induced ONFH [10]. Furthermore, we reported that ONFH results from the activation of nuclear factor kappa B (NF-κB) and interferon regulatory factor 7 (IRF7) via the TLR signaling pathways, followed by a subsequent repression in NF-κB activity by corticosteroid treatment, whereas IRF7 activity is unaffected by corticosteroid treatment. Further, the suppression of IRF7 activity by the use of an inhibitor, BAY11-7082, could interfere with the development of ONFH while maintaining the therapeutic effect of corticosteroids [9]. However, as rats co-administered with BAY11-7082 and corticosteroids showed high mortality, BAY11-7082 may be unsafe for human use. As new drug development requires an enormous amount of time and money, drug repositioning has been attracting a good deal of attention in recent years [11, 12].

The drug lansoprazole (LPZ) is a proton pump inhibitor used to treat and prevent stomach ulcers by suppressing acid secretion through proton pump inhibition [13]. It has, however, also been reported to have anti-inflammatory effects and suppress inflammatory responses via TLR4 signaling [14, 15].

In the present study, we hypothesized that LPZ has the potential to suppress IRF7 and NF-κB, in the same manner as BAY11-7082, and so prevent corticosteroid-induced ONFH in rats. Moreover, we conducted a preliminary clinical trial for the prevention of ONFH in patients with corticosteroid-treated immune diseases.

## Experimental materials and methods

### Animals

All experiments observed the guidelines of the Ministry of Sports, Culture, Science, and Technology of Japan, and followed protocols approved by the Animal Ethics Committee of Sapporo Medical University (#12-084). Male Wistar ST rats (300–350 g), obtained from Sankyo Labo Service Co., Ltd. (Sapporo, Japan), were housed in temperature- and humidity-controlled rooms with unlimited normal food and water and a 12-h light/dark cycle.

### Experimental groups and protocols

Animals (*n *= 80) were divided into four groups and treated, as in previous reports [8, 9], as follows: Lipopolysaccharide (LPS) + methylprednisolone (MPSL) rats (*n *= 18) were given 1.0 mg/kg LPS (from *Escherichia coli* serotype 055: B5; Sigma, St. Louis, MO, USA), a ligand for TLR4, intravenously on Day 1 and 20 mg/kg MPSL (Sigma, St. Louis, USA) intramuscularly on Day 2; LPS + LPZ + MPSL rats (*n *= 22) were given 1.0 mg/kg LPS intravenously on Day 1 and 5 mg/kg LPZ (Takepron^®^ intravenous 30 mg, Takeda Pharmaceutical Company Limited, Osaka, Japan) intravenously with 20 mg/kg MPSL intramuscularly on Day 2; imiquimod + MPSL rats (*n *= 18) were given 30 mg/kg imiquimod (Tokyo Chemical Industry, Tokyo, Japan), a ligand for TLR7, subcutaneously on Day 1 and 20 mg/kg MPSL intramuscularly on Day 2; and imiquimod + LPZ + MPSL rats (*n *= 22) were given 30 mg/kg imiquimod subcutaneously on Day 1 and 5 mg/kg LPZ intravenously with 20 mg/kg MPSL intramuscularly on Day 2. All injections were performed at 7:00 p.m.

Animals were killed at 1 or 14 days after the last injection. The femurs and livers were harvested and fixed with 10% formalin-0.1 M phosphate buffer (pH 7.4). A portion of the liver from each rat killed at 1 day after the last injection was harvested and stored at − 84 °C until analysis.

### Histopathology

Bone samples were decalcified with Kalkitox™ (Wako Pure Chemical Industries, Ltd., Osaka, Japan), neutralized with a 5% sodium sulfate buffer, and then processed for routine hematoxylin and eosin staining to assess ONFH. Osteonecrosis was defined as the diffuse presence of empty lacunae or pyknotic nuclei in osteocytes within the bone trabeculae, accompanied by surrounding bone marrow cell necrosis [8, 16, 17].

### Electrophoretic mobility shift assay (EMSA)

NF-κB and IRF7 activity was assessed by EMSA, as described previously [18]. Briefly, equal amounts of liver nuclear extract (2.0 mg of protein) were incubated for 1 h at room temperature with ^32^P-labeled NF-κB or IRF7 consensus oligonucleotide probes (5′-*AGTTGAGGGGACTTTCCCAGGC*-3′ or 5′-*ACTGATCGGAACCGAACGATCTATG*-3′, respectively) in binding buffer (10 mM HEPES [pH 7.9], 50 mM KCl, 0.2 mM ethylenediaminetetraacetic acid, 2.5 mM dithiothreitol, 10% glycerol, and 0.05% NP-40). The DNA protein complexes were separated on 7% non-denaturing polyacrylamide gels at a constant 100 V at room temperature. The gels were then exposed to an Image Plate (Fuji Film, Tokyo, Japan) at room temperature, and the radioactivity of their DNA-binding complexes was analyzed using an FLA3000 Image Analyzer (Fuji Film) and ImageQuant Software (Molecular Dynamics, Sunnyvale, CA, USA).

### Statistical analysis

Data represent the mean ± SEM. Comparisons between two groups were performed using 2-tailed Fisher’s exact test or the 2-tailed nonparametric Mann–Whitney test, using GraphPad Prism 6.0f software for Mac OS X (GraphPad Software, Inc., La Jolla, CA, USA). A *p* value < 0.05 was considered significant.

## Clinical patients and methods

### Patients

The study was approved by the Institutional Review Board of Sapporo Medical University Hospital (Approval number: #23-119) and observed the standards of the 1964 Declaration of Helsinki. Our study was a prospective, single-center, historically controlled trial. All patients required primary high-dose prednisolone treatment for immune diseases and were recruited in the departments of Gastroenterology, Rheumatology and Clinical Immunology of Sapporo Medical University Hospital in Sapporo, Japan, between July 2011 and September 2014. Inclusion criteria required a prednisolone dose of 35 mg/day or more and an age of 20–75 years. Exclusion criteria were as follows: current ONFH, hip joint disease requiring surgery, alcohol-abuse, dementia, past allergy to LPZ, and treatment with atazanavir sulfate. All 31 patients recruited provided written informed consent.

### Study procedure

All patients were administered LPZ (Takepron^®^ intravenous 30 mg, Takeda Pharmaceutical Company Limited, Osaka, Japan) intravenously a total of 6 times (once the night before corticosteroid treatment started, and thereafter twice a day). Subsequently, all were administered LPZ (Takepron^®^ OD 30 mg, Takeda Pharmaceutical Company Limited) orally once a day for 25 days. Routine magnetic resonance imaging (MRI) of the hips was performed before corticosteroid treatment, and at 4, 12 and 24 weeks thereafter using a GE Signa HDx 1.5 T (GE Healthcare, Milwaukee, WI, USA). T1-weighted images, T2-weighted images, and fat suppression images on the axial and coronal plane were obtained. A low signal intensity band on T1-weighted images was defined as ONFH. Two trained orthopedists and a trained radiologist assessed all radiographs. One patient was excluded because of a physical condition that precluded the MRI at 12 weeks. ONFH was diagnosed using the classifications for the osteonecrosis of the femoral head of the Japanese Ministry of Health, Labor, and Welfare [19], in which Type A lesion occupies the medial one-third or less of the weight-bearing portion, Type B lesion occupies the medial two-thirds or less of the weight-bearing portion, Type C1 and Type C2 lesions both occupy more than the medial two-thirds of the weight-bearing portion, with Type C2 lesions extending laterally to the acetabular edge, whereas Type C1 lesions do not. The margins of the necrotic areas were determined as a low signal intensity band at the coronal slice of the center of the femoral head on the T1-weighted images.

### Patient assessment

Table 1 shows patient demographic data including age, gender, underlying diseases, maximal daily prednisolone dosage, total prednisolone dosage within 3 months, days of corticosteroid treatment at 1000 mg/day, and occurrence of ONFH. The patients consisted of 17 men and 13 women (mean age 54.9 years). The underlying diseases were IgG4-related disease (*n *= 14), dermatomyositis (*n *= 8), systemic lupus erythematosus (SLE, *n *= 5), and others (*n *= 3). The maximal mean prednisolone dosage was 46.0 (35–60) mg/day, excluding 3 days of treatment at 1000 mg/day in two patients.

**Table 1:** Patient characteristics

	Age	Sex	UD	MD (mg/day)	TD (mg/3 M)	PD (days)	ONFH
1	20	F	SLE	50	3410	0	–/–
2	49	F	IgG4	40	2737.5	0	–/–
3	64	M	IgG4	40	2570	0	C-1/–
4	67	M	MPA	40	2770	0	–/–
5	57	M	IgG4	40	2737.5	0	C-1/B
6	58	M	IgG4	50	3207.5	0	–/–
7	70	M	IgG4	40	2757.5	0	–/–
8	65	M	DM	50	3580	0	–/–
9	25	M	AOSD	50	3370	0	–/–
10	48	F	SLE	50	3335	0	C-2/B
11	43	F	SLE	50	3990	0	–/–
12	68	M	IgG4	50	3335	0	–/–
13	62	M	IgG4	50	3250	0	–/–
14	64	M	IgG4	50	3365	0	–/–
15	50	M	IgG4	50	3340	0	–/–
16	58	F	IgG4	50	3140	0	–/–
17	63	M	SLE	60	3935	0	–/–
18	46	M	DM	40	2557.5	0	–/–
19	52	F	IgG4	40	2805	0	–/–
20	49	F	SS	35	2695	0	–/–
21	61	F	DM	40	2665	0	–/–
22	64	M	IgG4	40	2790	0	–/–
23	45	F	DM	60	4025	0	–/–
24	28	F	DM	50	3750	0	–/–
25	69	M	IgG4	40	2797.5	0	–/–
26	39	M	SLE	40	10,160	6	–/–
27	68	M	IgG4	40	2665	0	C-1/–
28	72	F	DM	50	3390	0	–/–
29	56	F	DM	35	2250	0	–/–
30	68	F	DM	50	6780	3	–/–

*UD* underlying disease, *MD* maximal corticosteroid dosage, *TD* total corticosteroid dosage within 3 months. *PD* days at 1000 mg/day

*SLE* systemic lupus erythematosus, *IgG4* IgG4-related disease, *MPA* microscopic polyangitis, *DM* dermatomyositis, *AOSD* Adult-onset Still’s disease, *SS* Sjogren’s syndrome, *ONFH* classification of the extent of the necrotic area was given

Owing to the lack of an effective nonsurgical treatment for ONFH, we did not conduct a randomized control study. We used a historical control group (14 men and 44 women, mean age 45.2 years) of patients from the same institute, who were the subject of a previous report [20]. Their underlying diseases were SLE (*n *= 16), IgG4-related disease (*n *= 11. Mikulicz’s disease, as described in previous reports), adult-onset Still’s disease (*n *= 8), dermatomyositis (*n *= 6), microscopic polyangitis (*n *= 6), and others (*n *= 11). The maximal mean prednisolone dosage was 45.2 mg/day, excluding 3 days of treatment at 1000 mg/day in ten patients. These parameters, excluding gender consistency (*p *< 0.01, Fisher’s exact test), were not significantly different compared to the LPZ treatment group (age: *p *= 0.06, Mann–Whitney test, underlying diseases: *p *= 0.09, Chi-square test, maximal steroid dosage: *p *= 0.76, Mann–Whitney test).

### Statistical analysis

.Comparisons between the two groups was performed with a 2-tailed Fisher’s exact test, Chi-square test or a 2-tailed nonparametric Mann–Whitney test using GraphPad Prism 6.0f software for Mac OS X (GraphPad Software, Inc., La Jolla, CA, USA). A *p* value < 0.05 was considered significant.

## Experimental results

**Fig. 1:**
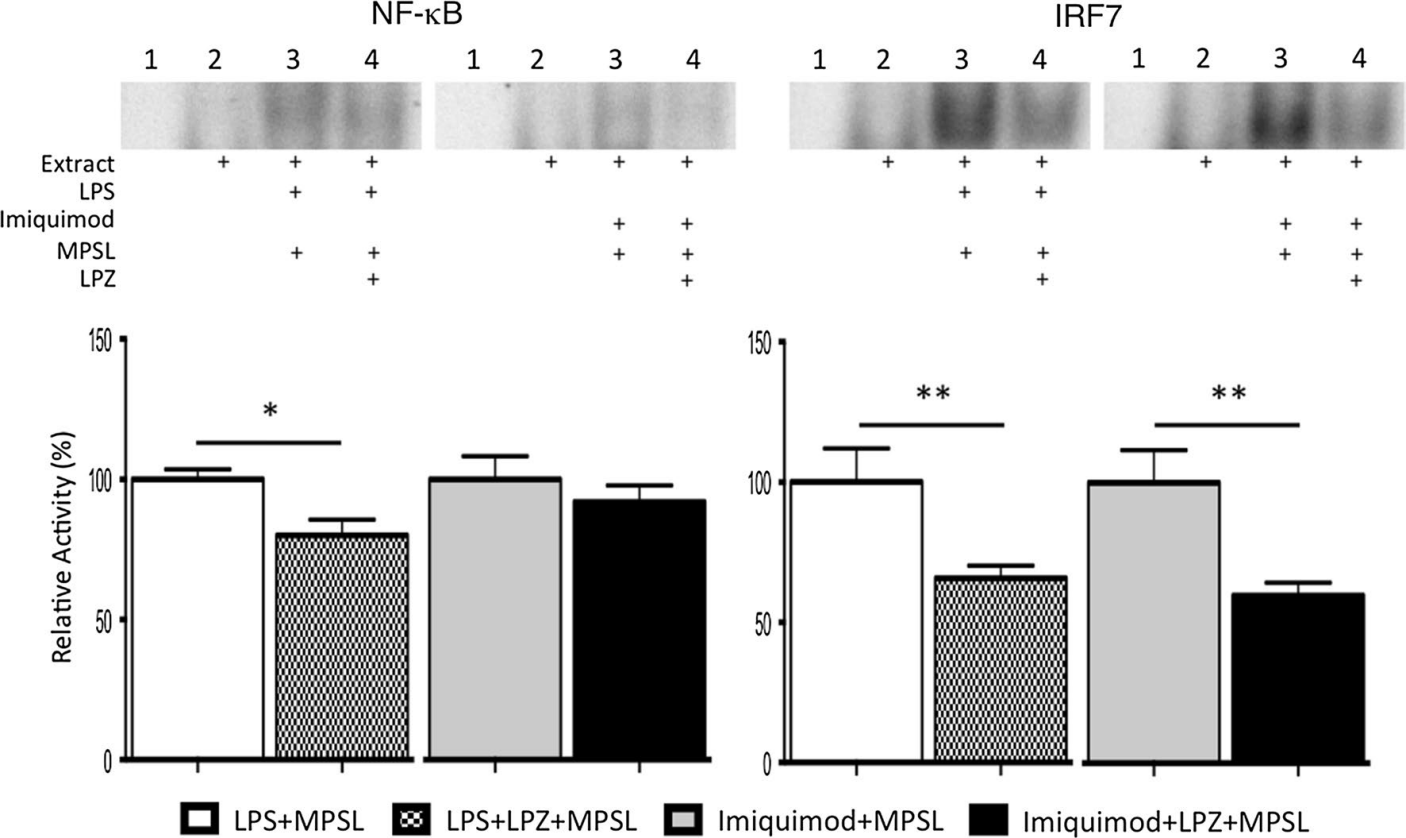
Transcription factor activity. EMSA for NF-κB and IRF7 in LPS + MPSL, LPS + LPZ + MPSL, imiquimod + MPSL and imiquimod + LPZ + MPSL rats on Day 1. Lane 1 contains no extract. Lane 2 contains extract from untreated rats. Lane 3 contains extract from rats treated with LPS/imiquimod + MPSL. Lane 4 contains extract from rats treated with LPS/imiquimod + LPZ + MPSL. A significant suppression of NF-κB activity was observed at Day 1 after the last injection in the LPS + LPZ + MPSL group compared with that in the LPS + MPSL group. A significant suppression in IRF7 activity was also observed at Day 1 after the co-administration of LPZ with corticosteroids. Data represent the mean ± SEM. *p < 0.05, **p < 0.01 compared with LPS/imiquimod + MPSL rats

To evaluate the activity of transcription factors NF-κB and IRF7 in the liver, the LPS + MPSL (*n *= 6) and imiquimod + MPSL (*n *= 6) (control groups) and LPS + LPZ + MPSL (*n* = 10) and imiquimod + LPZ + MPSL (*n *= 10) (experimental groups) rats were killed at 1 day after the last injection. Figure 1 shows the activity of transcription factors NF-κB and IRF7 in the liver. LPZ treatment significantly suppressed the activity of NF-κB in the LPS + LPZ + MPSL group compared to that in the LPS + MPSL group at 1 day after the last injection (Fig. 1: LPS + MPSL *vs.* LPS + LPZ + MPSL, 100 ± 3.6 *vs.* 80.5 ± 5.6; *p *= 0.03). However, there was no significant difference in NF-κB activity between the imiquimod + LPZ + MPSL and imiquimod + MPSL groups at that time (Fig. 1: imiquimod + MPSL *vs.* imiquimod + LPZ + MPSL, 100 ± 8.3 *vs.* 92.0 ± 5.9; *p *= 0.43). On the other hand, LPZ treatment significantly suppressed the activity of IRF7 in the LPS + LPZ + MPSL group compared to that in the LPS + MPSL and imiquimod + LPZ + MPSL groups as well as the imiquimod + MPSL group at 1 day after the last injection (Fig. 1: LPS + MPSL *vs.* LPS + LPZ + MPSL, 100 ± 11.94 *vs.* 65.7 ± 4.6; *p *= 0.02. Imiquimod + MPSL *vs.* imiquimod + LPZ + MPSL, 100 ± 11.7 *vs.* 59.8 ± 4.6; *p *= 0.02).

**Fig. 2:**
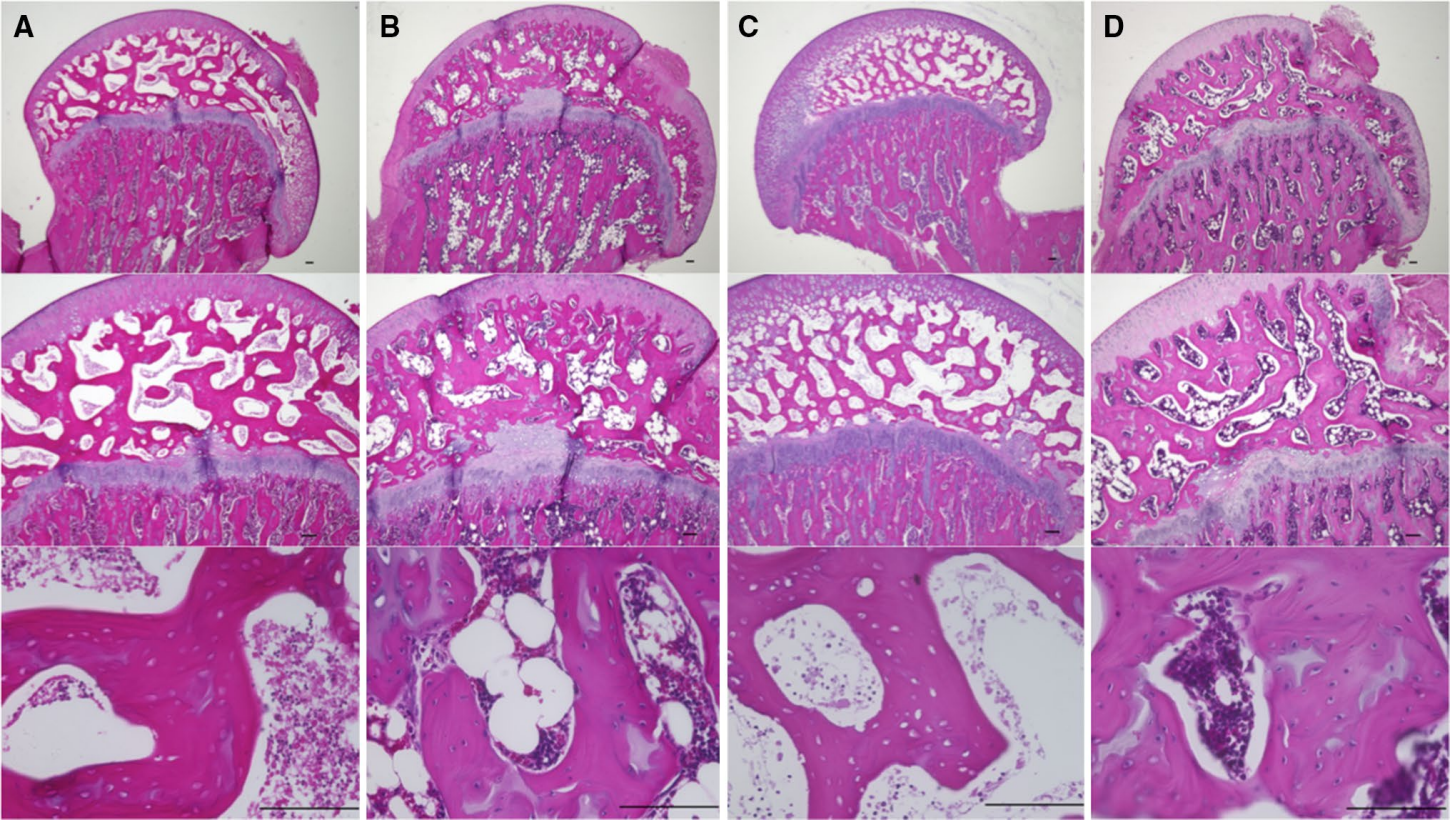
Histological appearance of the femoral head of rats killed at 14 days after the last injection. Panels show hematoxylin and eosin-stained femurs. Typical images from the LPS + MPSL (**a**), LPS + LPZ + MPSL (**b**), imiquimod + MPSL (**c**) and imiquimod + LPZ + MPSL (**d**) groups are shown. The diffuse presence of empty lacunae and pyknotic osteocytic nuclei in the bone trabeculae accompanied by bone marrow cell necrosis was observed in the femoral head in the LPS + MPSL and imiquimod + MPSL groups. Scale bar: 100 um

To evaluate the incidence of ONFH, the LPS + MPSL (*n *= 12) and imiquimod + MPSL (n = 12) (control groups) and LPS + LPZ + MPSL (*n *= 12) and imiquimod + LPZ + MPSL (*n *= 12) (experimental groups) rats were killed 14 days after the last injection. ONFH was observed in 5 of 11 (one had died prematurely) rats in the LPS + MPSL group and 6 of 12 rats in the imiquimod + MPSL group. By contrast, no ONFH was observed in the LPS + LPZ + MPSL (0 of 12) or imiquimod + LPZ + MPSL groups (0 of 12). The incidence in the LPZ-treated groups (0 of 24) was significantly lower than in the control groups (11/23) (*p *< 0.0001; Fisher’s exact test). Figure 2 shows the histopathological appearance of the femoral head after hematoxylin and eosin staining in the LPS + MPSL (A), LPS + LPZ + MPSL (B), imiquimod + MPSL (C) and imiquimod + LPZ + MPSL (D) rats. In the LPS + MPSL and imiquimod + MPSL groups, empty lacunae and pyknotic nuclei were observed within the necrotic bone trabeculae, and bone marrow cell necrosis was present in the medullary space across most areas of the femoral head (Fig. 2a, c). Normal trabeculae as well as hematopoietic and fat cells were observed in the LPS + LPZ + MPSL and imiquimod + LPZ + MPSL rats (Fig. 2b, d).

## Clinical results

As described above, we found that LPZ has the potential to prevent ONFH development in rats. Thus, we conducted a preliminary clinical trial.

In this clinical study, no adverse events associated with LPZ administration were observed. ONFH was found in 4 of 30 (13.3%) patients treated with high-dose corticosteroids, which was a significantly lower incidence (*p *= 0.0002; Fisher’s exact test) than that in the control group; i.e., 31 of 58 (53.4%). No low signal intensity band was observed on T1-weighted images pre-corticosteroid treatment or at 4 weeks after treatment. However, a band was observed in 3 patients at 12 weeks after treatment and in another patient at 24 weeks after treatment.

The patients with ONFH consisted of 3 males and one female, with mean age of 59.3 (48–68) years, and observation was continued in the Department of Orthopedic Surgery, Sapporo Medical University Hospital in Sapporo, Japan. The underlying diseases in the patients with ONFH were IgG4-related disease (*n *= 3, three males) and SLE (*n *= 1, female) (Table 1). The mean dosage of prednisolone over the 3-month period did not differ significantly in the patients with ONFH (Table 2; ONFH *vs*. Non-ONFH, 2827 ± 172.8 *vs*. 3571 ± 311.5; *p *= 0.073; Mann–Whitney test). The patients with ONFH did not receive 1000 mg/day corticosteroid treatment. Two developed bilateral ONFH, and two developed unilateral ONFH. According to the classifications of the osteonecrosis of the femoral head of the Japanese Ministry of Health, Labor, and Welfare, 2 patients developed type B, 3 patients type C-1, and 1 patient type C-2, with no collapse of the femoral head or other symptoms.

**Table 2:** Corticosteroid dosage

	~ 1 week	~ 2 weeks	~ 1 month	~ 2 months	~ 3 months
ONFH (*n *= 4)	297.5 ± 17.5	595.0 ± 35.0	1190 ± 70.0	2121 ± 108.2	2827 ± 172.8
Non-ONFH (*n *= 26)	325.8 ± 10.0	765.4 ± 111.9	1532 ± 165.0	2691 ± 283.1	3571 ± 311.5

**Fig. 3:**
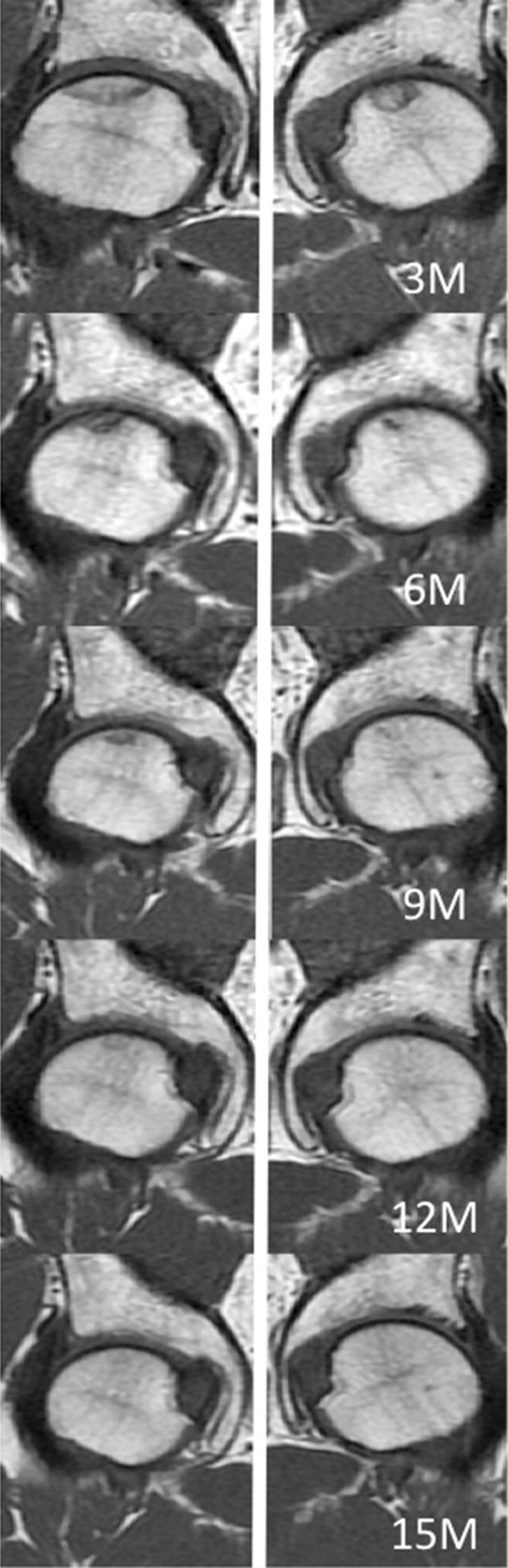
T1-weighted images of bilateral coronal MRI in an asymptomatic 57-year-old man. ONFH was detected at 3 months after corticosteroid treatment (Type C1 lesion in the right femoral head and Type B lesion in the left side). Six months after treatment, the ONFH lesion had decreased in size. The left femoral head ONFH lesion disappeared at 12 months after corticosteroid treatment and the right femoral head ONFH lesion disappeared at 15 months after corticosteroid treatment

In all patients, ONFH lesions with a low signal intensity band on T1 weighted images decreased in size during the observation period. A 57-year-old man with IgG4-related disease developed bilateral ONFH at 12 weeks after corticosteroid treatment. However, the ONFH lesions had become smaller in the MR images at 24 weeks. The left-side lesion disappeared at 12 months after corticosteroid treatment, and the right-side lesion at 15 months (Fig. 3). A 68-year-old man with IgG-related disease developed right-side ONFH at 24 weeks after corticosteroid treatment. However, the ONFH lesion had become smaller at 9 months, and observation was continued. A 64-year-old man with IgG-related disease developed right-side ONFH at 12 weeks after corticosteroid treatment. However, the ONFH lesion had become smaller at 14 months, and once again observation was continued. A 48-year-old woman with SLE developed right-side ONFH at 12 weeks and left-side ONFH at 24 weeks. However, the ONFH lesions had decreased in size at 24 months, and observation was continued. None of these patients received any special treatment such as avoidance of weight bearing.

## Discussion

ONFH leads to collapse of the femoral head in 80% of cases, results in osteoarthritis in young patients and affects the patient’s quality of life [7, 21]. The present study showed that the co-treatment of LPZ with corticosteroids prevents the development of ONFH in experimental animals, as well as in patients with immune disease treated with corticosteroids. We believe that this is the first report to describe the prevention of corticosteroid-induced ONFH development in a clinical trial based on experimental outcomes.

It has been reported that innate immune signaling via TLR pathways contributes to the pathogenesis of underlying diseases in patients with corticosteroid-induced ONFH [8, 9]. Consequently, we previously reported that corticosteroid treatment after TLR4, TLR7 or TLR9 ligand injection induces ONFH in Wistar rats and that ONFH results from the activation of NF-κB and IRF7 via the MyD88-dependent pathway, followed by a subsequent repression in NF-κB activity by corticosteroid treatment, whereas IRF7 activity is unaffected by corticosteroid treatment. Furthermore, suppression of IRF7 activity using BAY11-7082, Ikk-α inhibitor and Ikk-β inhibitor with the potential to inhibit NF-κB and IRF7 activity [22] interferes with the development of ONFH [9]. Thus, we believe that a proinflammatory response induced by corticosteroids leads to the development of ONFH. However, as the safety of BAY11-7082 has not been approved for humans, its clinical application is difficult. Therefore, we explored the use of an approved, commercially available drug, which has the potential to suppress IRF7 activity. It has been reported that LPZ exerts anti-inflammatory effects by suppressing the induction of inflammatory cytokines via the suppression of NF-κB activity [14]. Consequently, we hypothesized that LPZ also inhibits IRF7 activity and prevents ONFH development in rats, and this study showed that LPZ significantly suppressed IRF7 activity in the LPS + LPZ + MPSL and imiquimod + LPZ + MPSL groups compared with that in the LPS + MPSL and imiquimod + MPSL groups. This is consistent with previous reports on the effect of BAY11-7082 [9] and confirms that LPZ inhibits IRF7 activity. Further, NF-κB activity was significantly suppressed in the LPS + LPZ + MPSL group only. We previously reported that corticosteroid treatment suppressed NF-κB activity in imiquimod + MPSL rats compared with that in imiquimod + Saline rats [7]. This result indicates that LPS activates NF-κB activity more than does imiquimod. Therefore, NF-κB activity was significantly suppressed by LPZ treatment, and the co-treatment of LPZ with corticosteroids prevents the development of ONFH in rats. The results of the experimental study indicate that LPZ may also prevent the development of ONFH in patients with immune diseases needing corticosteroid treatment.

It has been reported that antihyperlipidemic, antiplatelet and antioxidant agents prevent the development of osteonecrosis in experimental animals [23–25]. Although clinical studies about prevention for ONFH by statin therapy and anti-coagulant were reported [26, 27], there are no standard preventive methods today. Unfortunately, no effective prevention strategy for ONFH has been clinically established. In the present study, LPZ also prevented the development of corticosteroid-induced ONFH in a clinical study. However, ONFH did develop in four patients, although not in any rats. Patients were administered with LPZ at the approved dosage, suggesting the necessity to reconsider the dosage and usage period of LPZ.

Additionally, the ONFH lesions in the four patients were found to decrease in size, as evaluated by MRI, during the observation period. Reports exist of ONFH lesions, observed by MRI, regressing spontaneously over several years [28–31]. These reports studied specific diseases, such as renal recipients, severe acute respiratory syndrome and SLE, so there is a possibility that specific treatments were provided for each group of patients. The present study could not determine whether the disappearance of ONFH was due to its natural course or the effects of LPZ, and ONFH patients need to be observed in a future clinical study.

Several limitations of our clinical study should be noted. First, it was unclear whether the pharmacological effect of LPZ as prevention agent for osteonecrosis in rat models can be transferred to humans and there have been no reports to date. Second, the sample size was small, and it was a single-center and historically controlled study. Further, there were differences in underlying diseases in this study group from those in past reports, particularly, as a large number of patients with IgG4-related disease were included in this study. This is due to the high rate of patients treated for IgG4-related disease in our hospital. Furthermore, there was some bias in terms of underlying diseases between the study and control groups. Last, we could not evaluate transcriptional factor activity in the patients. Nevertheless, our clinical trial did show a decrease in the incidence of ONFH in our institute.

## Conclusions

The present study shows that the co-treatment of LPZ with corticosteroids prevents the development of ONFH through the suppression of IRF7 activity in rats. Moreover, LPZ also prevents ONFH development in immune disease patients treated with corticosteroids, although the study was only preliminary. Consequently, it is necessary to conduct a multicenter clinical trial with large sample size. Nevertheless, we believe that LPZ is safe to use and could be effective in preventing osteonecrosis of the femoral head in patients needing corticosteroid treatment. We anticipate that further clinical trials will show that this protocol is both consistent and reproducible.
